# The genome sequence of the giant clam, *Tridacna gigas *(Linnaeus, 1758)

**DOI:** 10.12688/wellcomeopenres.21136.1

**Published:** 2024-03-19

**Authors:** Ruiqi Li, Jingchun Li, Jose Victor Lopez, Graeme Oatley, Isabelle Ailish Clayton-Lucey, Elizabeth Sinclair, Eerik Aunin, Noah Gettle, Camilla Santos, Michael Paulini, Haoyu Niu, Victoria McKenna, Rebecca O’Brien, Wellcome Sanger Institute Tree of Life Management, Samples and Laboratory Team, Wellcome Sanger Institute Scientific Operations: Sequencing Operations, Wellcome Sanger Institute Tree of Life Core Informatics Team, EBI Aquatic Symbiosis Genomics Data Portal Team, Aquatic Symbiosis Genomics Project Leadership

**Affiliations:** 1 Ecology & Evolutionary Biology, University of Colorado Boulder, Boulder, Colorado, USA; 2 Museum of Natural History, University of Colorado Boulder, Boulder, Colorado, USA; 3 Department of Biological Sciences, Nova Southeastern University, Dania Beach, Florida, USA; 4 Tree of Life, Wellcome Sanger Institute, Hinxton, England, UK

**Keywords:** Tridacna gigas, giant clam, genome sequence, chromosomal, Veneroida

## Abstract

We present a chromosomal-level genome assembly from an individual *Tridacna gigas* (the giant clam; Mollusca; Bivalvia; Veneroida; Cardiidae). The genome sequence is 1,175.9 megabases in span. Most of the assembly is scaffolded into 17 chromosomal pseudomolecules. The mitochondrial genome has also been assembled and is 25.34 kilobases in length. Gene annotation of this assembly on Ensembl identified 18,177 protein coding genes.

## Species taxonomy

Eukaryota; Opisthokonta; Metazoa; Eumetazoa; Bilateria; Protostomia; Spiralia; Lophotrochozoa; Mollusca; Bivalvia; Autobranchia; Heteroconchia; Euheterodonta; Imparidentia; Neoheterodontei; Cardiida; Cardioidea; Cardiidae; Tridacninae; Tridacna; *Tridacna gigas* (Linnaeus, 1758) (NCBI:txid80829).

## Background

Giant clams (subfamily Tridacninae) are the largest extant bivalves ( Soo & Todd, 2014). All species within the subfamily form a photosymbiotic partnership with Symbiodiniaceae dinoflagellates ( Ip & Chew, 2021). In addition to their reef building capacity, giant clams serve as reservoirs of Symbiodiniaceae, offer substrates for epibionts to colonise, and enhance coral reefs’ topographic heterogeneity ( Neo *et al.*, 2015). Among the twelve currently recognised extant species, *Tridacna gigas* is a true gigantic species, with the largest individual measuring an impressive 137 cm in length and weighing a remarkable 500 kg ( Neo, 2023).

*T. gigas* naturally distribute in shallow tropical habitats in the central Indo-Pacific, ranging from Myanmar to Kiribati, and Ryukyus to Queensland ( Neo *et al.*, 2017). Due to its enormous size, it faces extensive exploitation from over-fishing for both its flesh and shells, and increasing demands from the aquarium trade, despite CITES regulations ( Tan *et al.*, 2022). Coupled with the effects of global warming and ocean acidification, *T. gigas* populations have been declining rapidly in the wild, and many failed to recover ( Gomez, 2015).

Examining the chromosome-level genome assembly of *T. gigas* allows us to gain deeper insights into its population demographics, and the genetic framework that underlies the symbiotic relationship with Symbiodiniaceae, which may lead to practical conservation strategies during this era of climate change. Conducting comparative genomics analyses among various giant clam species may also uncover genetic mechanisms responsible for the remarkable size of *T. gigas*. Being part of the broader Aquatic Symbiosis Genomics project ( McKenna *et al.*, 2021), which includes sequencing diverse photosymbiotic hosts, we have the opportunity to explore both shared and novel molecular pathways in different species and gain comprehensive understanding of the evolution of photosymbiosis.

## Genome sequence report

**Figure 1.**
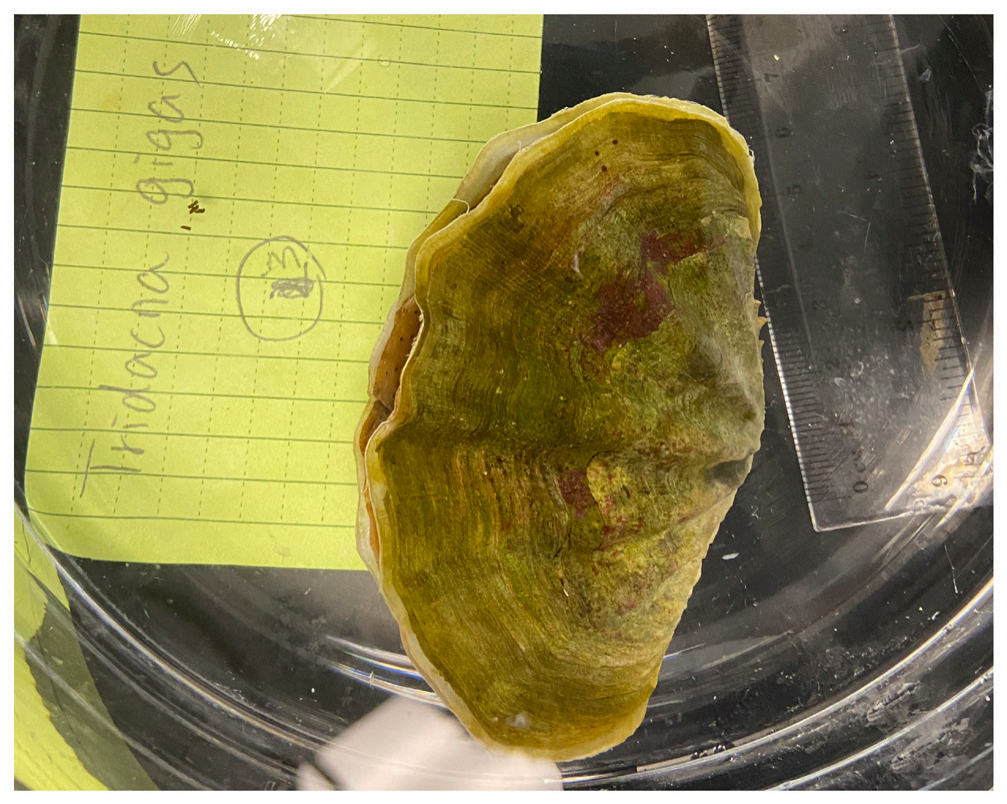
Photograph of the *Tridacna gigas* (xbTriGiga4) specimen used for genome sequencing.

The genome was sequenced from a specimen of *Tridacna gigas* ( Figure 1) collected from Marshall Islands Mariculture Farm, Majuro, Marshall Islands. A total of 36-fold coverage in Pacific Biosciences single-molecule HiFi long reads was generated. Primary assembly contigs were scaffolded with chromosome conformation Hi-C data. Manual assembly curation corrected 29 missing joins or mis-joins and removed 23 haplotypic duplications, reducing the assembly length by 0.71% and the scaffold number by 55.32.

**Figure 2.**
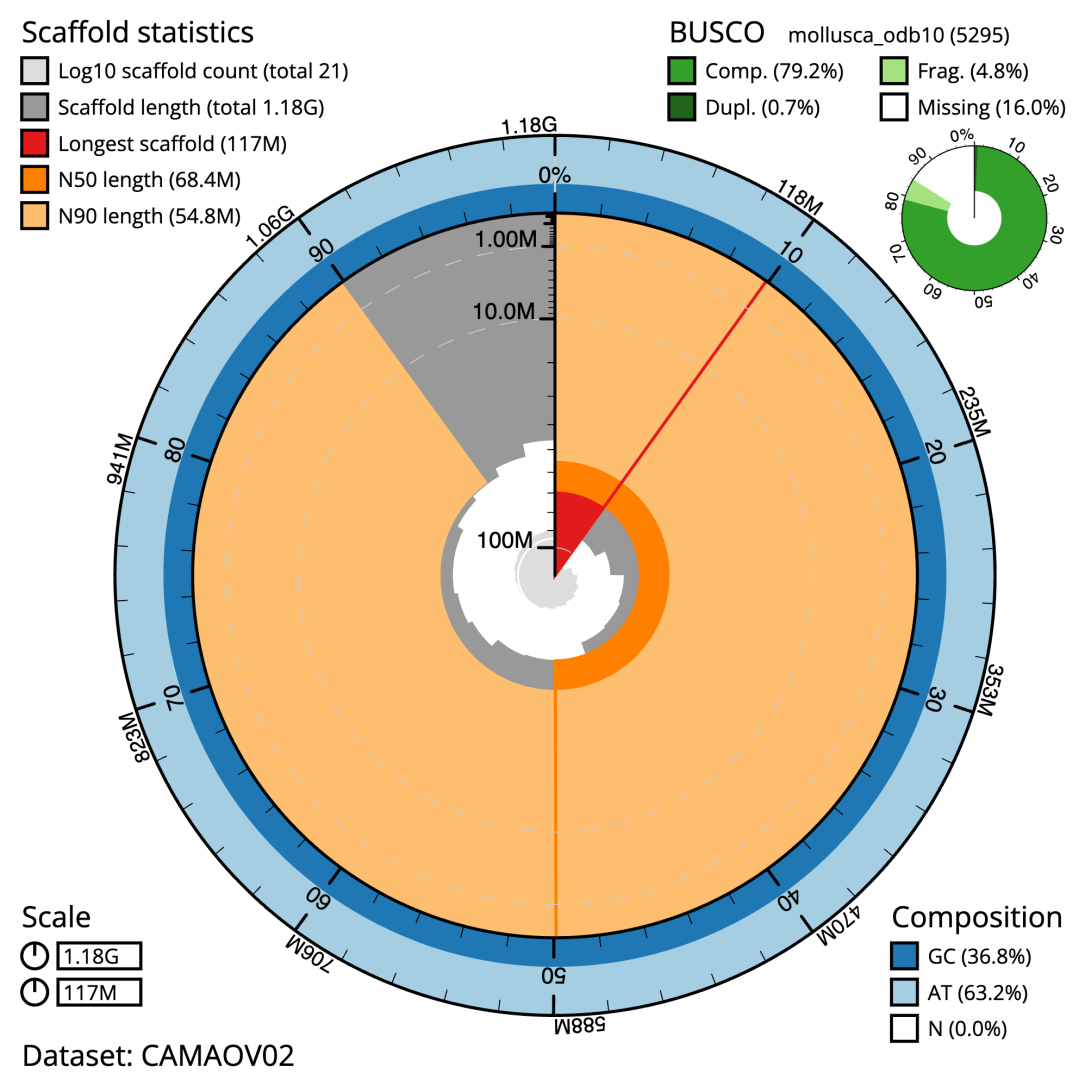
Genome assembly of *Tridacna gigas*, xbTriGiga4.2: metrics. The BlobToolKit snail plot shows N50 metrics and BUSCO gene completeness. The main plot is divided into 1,000 size-ordered bins around the circumference with each bin representing 0.1% of the 1,175,968,439 bp assembly. The distribution of scaffold lengths is shown in dark grey with the plot radius scaled to the longest scaffold present in the assembly (117,261,666 bp, shown in red). Orange and pale-orange arcs show the N50 and N90 scaffold lengths (68,447,427 and 54,827,388 bp), respectively. The pale grey spiral shows the cumulative scaffold count on a log scale with white scale lines showing successive orders of magnitude. The blue and pale-blue area around the outside of the plot shows the distribution of GC, AT and N percentages in the same bins as the inner plot. A summary of complete, fragmented, duplicated and missing BUSCO genes in the mollusca_odb10 set is shown in the top right. An interactive version of this figure is available at https://blobtoolkit.genomehubs.org/view/CAMAOV02/dataset/CAMAOV02/snail.

**Figure 3.**
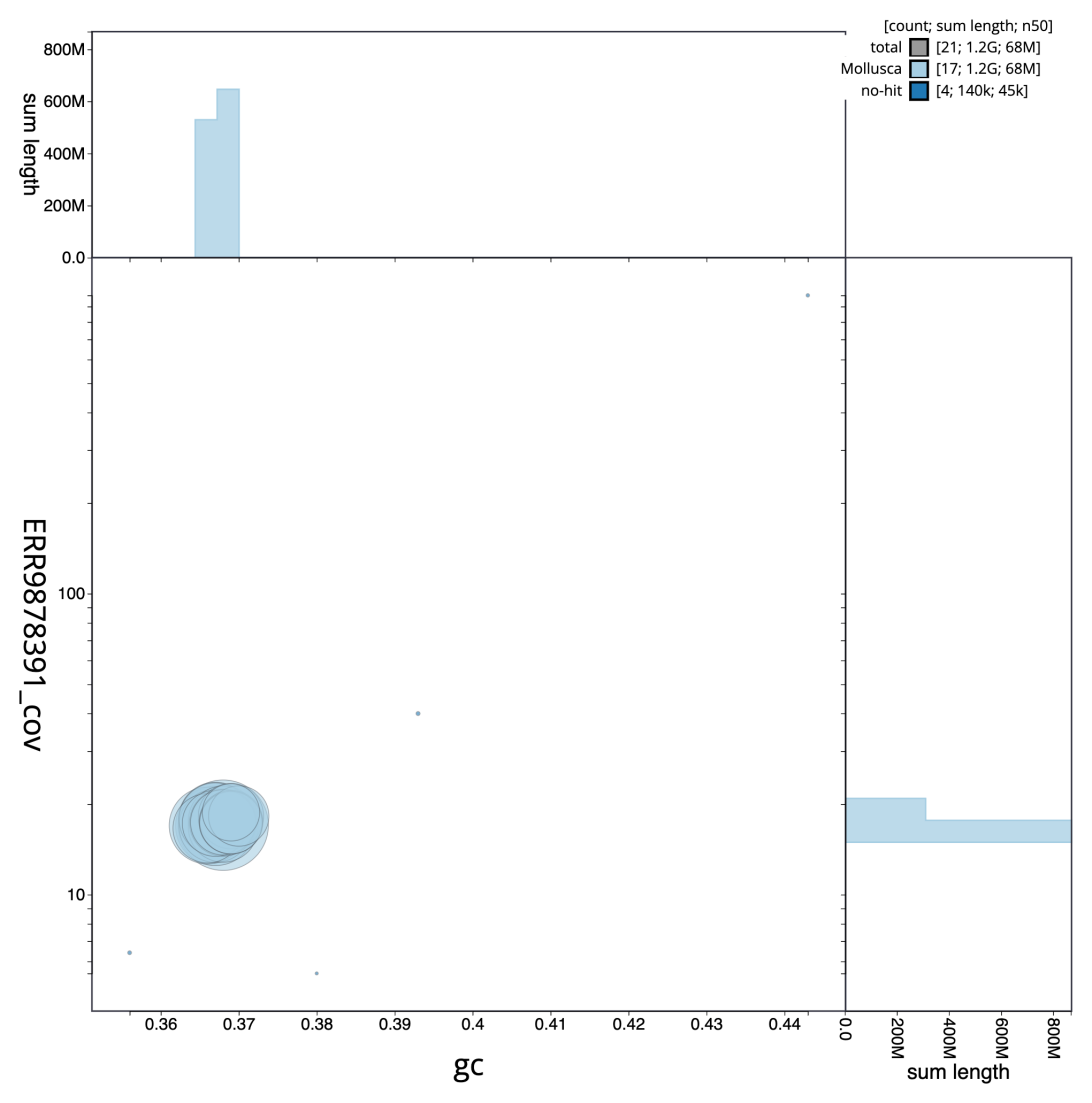
Genome assembly of *Tridacna gigas*, xbTriGiga4.2: BlobToolKit GC-coverage plot. Scaffolds are coloured by phylum. Circles are sized in proportion to scaffold length. Histograms show the distribution of scaffold length sum along each axis. An interactive version of this figure is available at https://blobtoolkit.genomehubs.org/view/CAMAOV02/dataset/CAMAOV02/blob.

**Figure 4.**
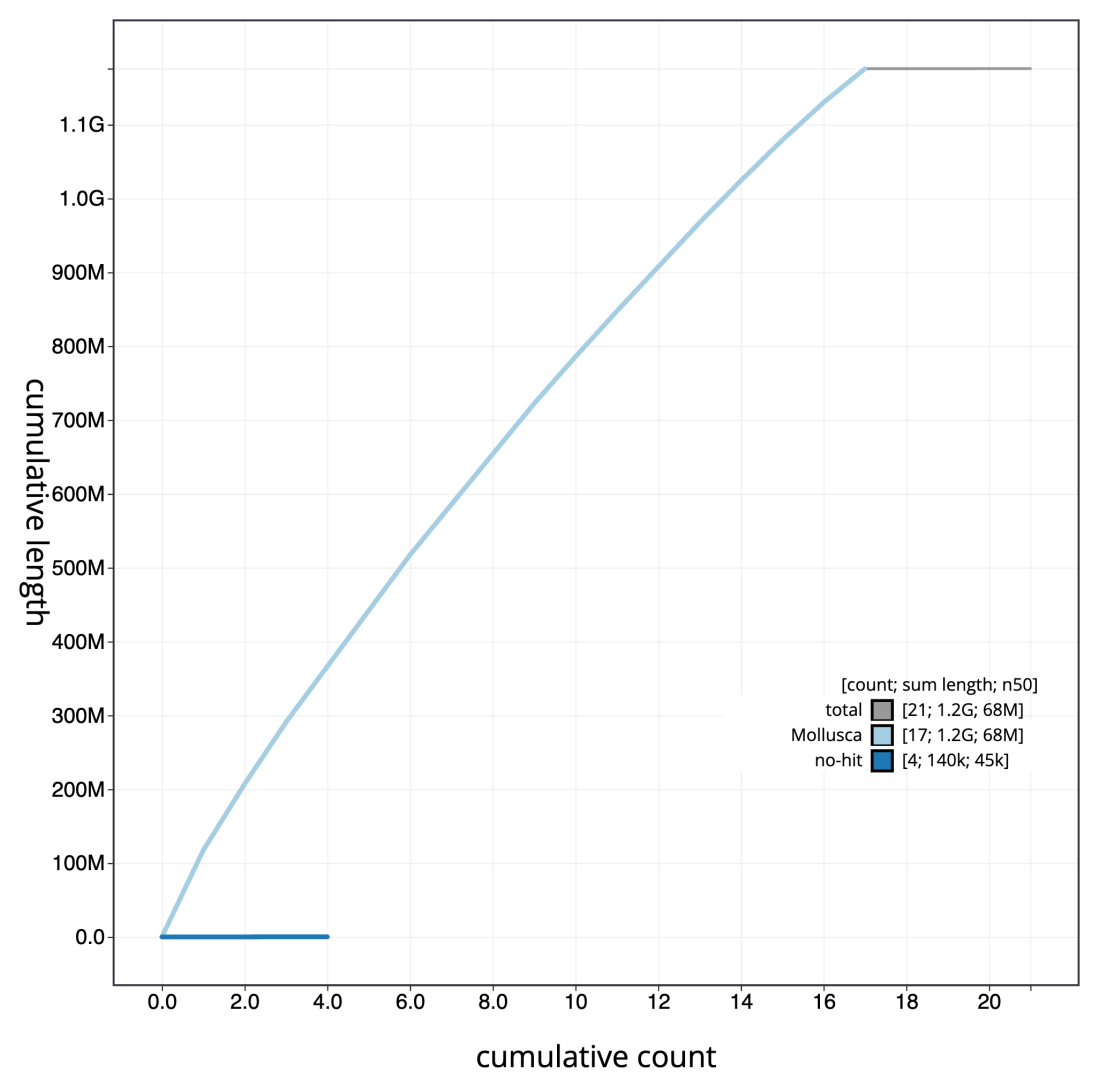
Genome assembly of *Tridacna gigas*, xbTriGiga4.2: BlobToolKit cumulative sequence plot. The grey line shows cumulative length for all scaffolds. Coloured lines show cumulative lengths of scaffolds assigned to each phylum using the buscogenes taxrule. An interactive version of this figure is available at https://blobtoolkit.genomehubs.org/view/CAMAOV02/dataset/CAMAOV02/cumulative.

**Figure 5.**
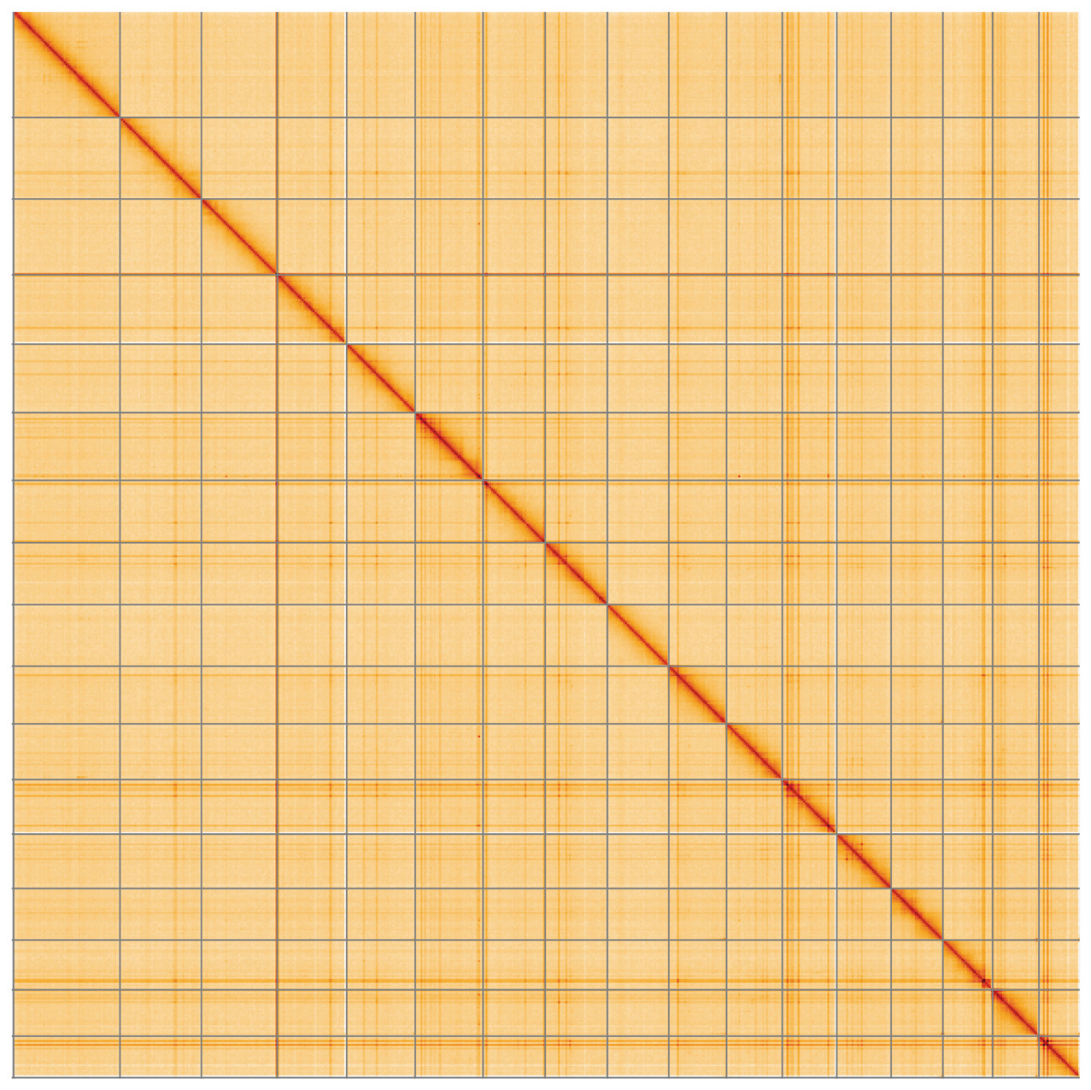
Genome assembly of *Tridacna gigas*, xbTriGiga4.2: Hi-C contact map of the xbTriGiga4.2 assembly, visualised using HiGlass. Chromosomes are shown in order of size from left to right and top to bottom. An interactive version of this figure may be viewed at https://genome-note-higlass.tol.sanger.ac.uk/l/?d=BscNBFj0TFu9wH4hMpdhvw.

The final assembly has a total length of 1175.9 Mb in 20 sequence scaffolds with a scaffold N50 of 68.4 Mb ( Table 1). The snail plot in Figure 2 provides a summary of the assembly statistics, while the distribution of assembly scaffolds on GC proportion and coverage is shown in Figure 3. The cumulative assembly plot in Figure 4 shows curves for subsets of scaffolds assigned to different phyla. Most (99.98%) of the assembly sequence was assigned to 17 chromosomal-level scaffolds. Chromosome-scale scaffolds confirmed by the Hi-C data are named in order of size ( Figure 5; Table 2). While not fully phased, the assembly deposited is of one haplotype. Contigs corresponding to the second haplotype have also been deposited. The mitochondrial genome was also assembled and can be found as a contig within the multifasta file of the genome submission.

**Table 1.** Genome data for *Tridacna gigas*, xbTriGiga4.2.

Project accession data		
Assembly identifier	xbTriGiga4.2	
Species	*Tridacna gigas*	
Specimen	xbTriGiga4	
NCBI taxonomy ID	80829	
BioProject	PRJEB53735	
BioSample ID	SAMEA8576962	
Isolate information	xbTriGiga4 (DNA, Hi-C and RNA sequencing)	
Assembly metrics *		*Benchmark*
Consensus quality (QV)	63.1	*≥ 50*
*k*-mer completeness	100.0%	*≥ 95%*
BUSCO **	C:79.2%[S:78.5%,D:0.7%], F:4.8%,M:16.0%,n:5,295	*C ≥ 95%*
Percentage of assembly mapped to chromosomes	99.98%	*≥ 95%*
Sex chromosomes	None	*localised homologous pairs*
Organelles	Mitochondrial genome: 25.34 kb	*complete single alleles*
Raw data accessions		
PacificBiosciences SEQUEL II	ERR9878391, ERR9878392	
Hi-C Illumina	ERR9881695	
PolyA RNA-Seq Illumina	ERR10378018	
Genome assembly		
Assembly accession	GCA_945859785.2	
*Accession of alternate haplotype*	GCA_945859735.2	
Span (Mb)	1,175.9	
Number of contigs	198	
Contig N50 length (Mb)	9.4	
Number of scaffolds	20	
Scaffold N50 length (Mb)	68.4	
Longest scaffold (Mb)	117.26	
Genome annotation		
Number of protein-coding genes	18,177	
Number of non-coding genes	6,818	
Number of gene transcripts	37,598	

* Assembly metric benchmarks are adapted from column VGP-2020 of “Table 1: Proposed standards and metrics for defining genome assembly quality” from Rhie *et al.* (2021). ** BUSCO scores based on the mollusca_odb10 BUSCO set using version 5.3.2. C = complete [S = single copy, D = duplicated], F = fragmented, M = missing, n = number of orthologues in comparison. A full set of BUSCO scores is available at https://blobtoolkit.genomehubs.org/view/CAMAOV02/dataset/CAMAOV02/busco.

**Table 2.** Chromosomal pseudomolecules in the genome assembly of *Tridacna gigas*, xbTriGiga4.

INSDC accession	Chromosome	Length (Mb)	GC%
OX244028.2	1	117.26	37.0
OX244029.2	2	89.79	36.5
OX244030.2	3	83.66	36.5
OX244031.2	4	76.43	36.5
OX244032.2	5	75.56	36.5
OX244033.2	6	74.72	36.5
OX244034.2	7	68.6	37.0
OX244035.2	8	68.45	37.0
OX244036.2	9	67.81	36.5
OX244037.2	10	63.53	37.0
OX244038.2	11	61.54	36.5
OX244039.2	12	60.02	37.0
OX244040.2	13	59.94	37.0
OX244041.2	14	56.93	37.0
OX244042.2	15	54.83	37.0
OX244043.2	16	51.05	37.0
OX244044.2	17	45.73	37.0
OX244045.2	MT	0.03	44.5

The estimated Quality Value (QV) of the final assembly is 63.1 with *k*-mer completeness of 100.0%, and the assembly has a BUSCO v5.3.2 completeness of 79.2% (single = 78.5%, duplicated = 0.7%), using the mollusca_odb10 reference set ( *n* = 5,295).

Metadata for specimens, barcode results, spectra estimates, sequencing runs, contaminants and pre-curation assembly statistics are given at https://links.tol.sanger.ac.uk/species/80829.

## Genome annotation report

The *Tridacna gigas* genome was annotated at the European Bioinformatics Institute (EBI) using the Ensembl rapid annotation pipeline ( Table 1; https://rapid.ensembl.org/Tridacna_gigas_GCA_945859785.2/Info/Index). The resulting annotation includes 37,598 transcribed mRNAs from 18,177 protein-coding and 6,818 non-coding genes.

## Methods

### Sample acquisition and nucleic acid extraction

A *Tridacna gigas* (specimen ID NSU0010103, ToLID xbTriGiga4) was purchased from Oceans, Reefs & Aquariums (ORA) in Marshall Islands Mariculture Farm, Majuro, Marshall Islands. The specimen was collected and identified by Jingchun Li and Ruiqi Li (University of Colorado Boulder), and then preserved by snap-freezing.

The workflow for high molecular weight (HMW) DNA extraction at the Wellcome Sanger Institute (WSI) includes a sequence of core procedures: sample preparation; sample homogenisation, DNA extraction, fragmentation, and clean-up. In sample preparation, the xbTriGiga4 sample was weighed and dissected on dry ice ( Jay *et al.*, 2023). For sample homogenisation, tissue was cryogenically disrupted using the Covaris cryoPREP ^®^ Automated Dry Pulverizer ( Narváez-Gómez *et al.*, 2023). HMW DNA was extracted using the Manual MagAttract v1 protocol ( Strickland *et al.*, 2023b). DNA was sheared into an average fragment size of 12–20 kb in a Megaruptor 3 system with speed setting 30 ( Todorovic *et al.*, 2023). Sheared DNA was purified by solid-phase reversible immobilisation ( Strickland *et al.*, 2023a): in brief, the method employs a 1.8X ratio of AMPure PB beads to sample to eliminate shorter fragments and concentrate the DNA. The concentration of the sheared and purified DNA was assessed using a Nanodrop spectrophotometer and Qubit Fluorometer and Qubit dsDNA High Sensitivity Assay kit. Fragment size distribution was evaluated by running the sample on the FemtoPulse system.

RNA was extracted from tissue of xbTriGiga4 in the Tree of Life Laboratory at the WSI using the RNA Extraction: Automated MagMax™ *mir*Vana protocol ( do Amaral *et al.*, 2023). The RNA concentration was assessed using a Nanodrop spectrophotometer and a Qubit Fluorometer using the Qubit RNA Broad-Range Assay kit. Analysis of the integrity of the RNA was done using the Agilent RNA 6000 Pico Kit and Eukaryotic Total RNA assay.

Protocols developed by the WSI Tree of Life laboratory are publicly available on protocols.io ( Denton *et al.*, 2023).

### Sequencing

Pacific Biosciences HiFi circular consensus DNA sequencing libraries were constructed according to the manufacturers’ instructions. Poly(A) RNA-Seq libraries were constructed using the NEB Ultra II RNA Library Prep kit. DNA and RNA sequencing was performed by the Scientific Operations core at the WSI on Pacific Biosciences SEQUEL II (HiFi) and Illumina NovaSeq 6000 (RNA-Seq) instruments. Hi-C data were also generated from tissue of xbTriGiga4 using the Arima2 kit and sequenced on the Illumina NovaSeq 6000 instrument.

### Genome assembly, curation and evaluation

Assembly was carried out with Hifiasm ( Cheng *et al.*, 2021) and haplotypic duplication was identified and removed with purge_dups ( Guan *et al.*, 2020). The assembly was then scaffolded with Hi-C data ( Rao *et al.*, 2014) using YaHS ( Zhou *et al.*, 2023). The assembly was checked for contamination and corrected using the gEVAL system ( Chow *et al.*, 2016) as described previously ( Howe *et al.*, 2021). Manual curation was performed using gEVAL, HiGlass ( Kerpedjiev *et al.*, 2018) and PretextView ( Harry, 2022). The mitochondrial genome was assembled using MitoHiFi ( Uliano-Silva *et al.*, 2023), which runs MitoFinder ( Allio *et al.*, 2020) or MITOS ( Bernt *et al.*, 2013) and uses these annotations to select the final mitochondrial contig and to ensure the general quality of the sequence.

A Hi-C map for the final assembly was produced using bwa-mem2 ( Vasimuddin *et al.*, 2019) in the Cooler file format ( Abdennur & Mirny, 2020). To assess the assembly metrics, the *k*-mer completeness and QV consensus quality values were calculated in Merqury ( Rhie *et al.*, 2020). This work was done using Nextflow ( Di Tommaso *et al.*, 2017) DSL2 pipelines “sanger-tol/readmapping” ( Surana *et al.*, 2023a) and “sanger-tol/genomenote” ( Surana *et al.*, 2023b). The genome was analysed within the BlobToolKit environment ( Challis *et al.*, 2020) and BUSCO scores ( Manni *et al.*, 2021; Simão *et al.*, 2015) were calculated.

Table 3 contains a list of relevant software tool versions and sources.

**Table 3.** Software tools: versions and sources.

Software tool	Version	Source
BlobToolKit	4.2.1	https://github.com/blobtoolkit/blobtoolkit
BUSCO	5.3.2	https://gitlab.com/ezlab/busco
gEVAL	N/A	https://geval.org.uk/
Hifiasm	0.16.1-r375	https://github.com/chhylp123/hifiasm
HiGlass	1.11.6	https://github.com/higlass/higlass
Merqury	MerquryFK	https://github.com/thegenemyers/MERQURY.FK
MitoHiFi	2	https://github.com/marcelauliano/MitoHiFi
PretextView	0.2	https://github.com/wtsi-hpag/PretextView
purge_dups	1.2.3	https://github.com/dfguan/purge_dups
sanger-tol/genomenote	v1.0	https://github.com/sanger-tol/genomenote
sanger-tol/readmapping	1.1.0	https://github.com/sanger-tol/readmapping/tree/1.1.0
YaHS	yahs-1.1.91eebc2	https://github.com/c-zhou/yahs

### Genome annotation

The Ensembl Genebuild annotation system ( Aken *et al.*, 2016) at the EBI was used to generate annotation for the *Tridacna gigas* assembly (GCA_945859785.2). Annotation was created primarily through alignment of transcriptomic data to the genome, with gap filling via protein-to-genome alignments of a select set of proteins from UniProt (UniProt Consortium, 2019).

### Wellcome Sanger Institute – Legal and Governance

The materials that have contributed to this genome note have been supplied by a Tree of Life collaborator. The Wellcome Sanger Institute employs a process whereby due diligence is carried out proportionate to the nature of the materials themselves, and the circumstances under which they have been/are to be collected and provided for use. The purpose of this is to address and mitigate any potential legal and/or ethical implications of receipt and use of the materials as part of the research project, and to ensure that in doing so we align with best practice wherever possible. The overarching areas of consideration are:

•     Ethical review of provenance and sourcing of the material

•     Legality of collection, transfer and use (national and international)

Each transfer of samples is undertaken according to a Research Collaboration Agreement or Material Transfer Agreement entered into by the Tree of Life collaborator, Genome Research Limited (operating as the Wellcome Sanger Institute) and in some circumstances other Tree of Life collaborators.

## Data Availability

European Nucleotide Archive: *Tridacna gigas* (giant clam). Accession number PRJEB53735; https://identifiers.org/ena.embl/PRJEB53735 ( Wellcome Sanger Institute, 2023). The genome sequence is released openly for reuse. The *Tridacna gigas* BioProject is part of the Aquatic Symbiosis Genomics (ASG) project ( PRJEB43743). All raw sequence data and the assembly have been deposited in INSDC databases. Raw data and assembly accession identifiers are reported in Table 1.

## Author information

Members of the Wellcome Sanger Institute Tree of Life Management, Samples and Laboratory Team are listed here: https://doi.org/10.5281/zenodo.10066175.

Members of the Wellcome Sanger Institute Scientific Operations: Sequencing Operations are listed here: https://doi.org/10.5281/zenodo.10043364.

Members of the Wellcome Sanger Institute Tree of Life Core Informatics team are listed here: https://doi.org/10.5281/zenodo.10066637.

Members of the European Bioinformatics Institute ASG Data Portal team are listed here: https://doi.org//10.5281/zenodo.10076466.

Members of the Wellcome Sanger Institute/Aquatic Symbiosis Genomics Project Leadership are listed here: https://doi.org/10.5281/zenodo.10184833.
