# Supplementary material for: The genome sequence of the giant clam, Tridacna gigas (Linnaeus, 1758)
Source: Wellcome Open Res. 2024 Mar 19;9:145. [Version 1] doi: 10.12688/wellcomeopenres.21136.1 (PMC11116938; doi:10.12688/wellcomeopenres.21136.1)
Supplement: Peer review history

# Reviewer report: Reviewer response for version 1

**DOI:** 10.21956/wellcomeopenres.23380.r79025

**Published:** 2024-05-23

**Authors:** Haitao Ma

**Affiliations:** 1 Chinese Academy of Sciences, Guangzhou, China

In this study, the whole genome sequencing and gene annotation of the *Tridacna gigas* were carried out. The results of this study will help us to gain deeper insights into its population demographics, and the genetic framework that underlies the symbiotic relationship with Symbiodiniaceae, which may lead to practical conservation strategies during this era of climate change. But there is the following small problem which need attention: As far as I know, through karyotype analysis and previous research results (Li *et al.*, 2024 ^1^, Zhang *et al.*, 2024 ^2^), it was found that the number of chromosomes of *Tridacna crocea* and *Tridacna squamosa* was 18 pairs of chromosomes. Why were only 17 chromosomal pseudomolecules obtained in this study?

Are sufficient details of methods and materials provided to allow replication by others?

Yes

Is the rationale for creating the dataset(s) clearly described?

Yes

Are the datasets clearly presented in a useable and accessible format?

Yes

Are the protocols appropriate and is the work technically sound?

Yes

Reviewer Expertise:

Marine Biology; Evolutionary Biology; Population Genetics; Genetic Breeding

I confirm that I have read this submission and believe that I have an appropriate level of expertise to confirm that it is of an acceptable scientific standard, however I have significant reservations, as outlined above.

## References

[1] : Chromosome-level genome assembly and annotation of rare and endangered tropical bivalve, Tridacna crocea. *Sci Data* .2024;11(1) : 10.1038/s41597-024-03014-8 186 10.1038/s41597-024-03014-8 38341475 PMC10858879

[2] : Genomic insights into photosymbiotic evolution inTridacna squamosa. *bioRxiv* .2024; 10.1101/2024.02.04.577604 10.1101/2024.02.04.577604

# Reviewer report: Reviewer response for version 1

**DOI:** 10.21956/wellcomeopenres.23380.r81945

**Published:** 2024-05-23

**Authors:** Charles Plessy

**Affiliations:** 1 Okinawa Institute of Science and Technology, Okinawa, Japan

The article is clear and follows the same pattern as the other works published here by the authors on other clam shells, which makes it easy to assess.

 The number of missing BUSCOs appears to be high (16%), but is comparable to the other Tridacninae chromosomal assemblies already reported in this journal. This said, it may be useful to rule out incompleteness of the assembly by also searching for BUSCOs in the transcriptome and showing that the missing ones are the same.  Alternatively, it could be checked if most missing BUSCOs are absent from both haplotypes, or also absent in other Tridacninae chromosomal assemblies.

Are sufficient details of methods and materials provided to allow replication by others?

Yes

Is the rationale for creating the dataset(s) clearly described?

Yes

Are the datasets clearly presented in a useable and accessible format?

Yes

Are the protocols appropriate and is the work technically sound?

Yes

Reviewer Expertise:

Pairwise genome comparisons

I confirm that I have read this submission and believe that I have an appropriate level of expertise to confirm that it is of an acceptable scientific standard.

# Reviewer report: Reviewer response for version 1

**DOI:** 10.21956/wellcomeopenres.23380.r81946

**Published:** 2024-05-21

**Authors:** Daniel Garcia-Souto

**Affiliations:** 1 University of Santiago de Compostela, Santiago de Compostela, Galicia, Spain

The authors present a much-needed genome assembly of the giant clam *Tridacna gigas*. This assembly was achieved at chromosome level, displaying top-tier annotation and completeness stats. All data is freely available well ahead of publication. This represents a significant addition for future genomics and comparative analysis.

 As a side note, from a pure taxonomical perspective, it would be beneficial to supplement these (and other) reports with more detailed views or photographs of the specimen. In addition to the general view of the animal, including the typical shell features used for species identification (such as hinge or pallial lines) would be highly valuable. This is especially important as bivalves can involve cryptic species or subspecies that may complicate identification.

Are sufficient details of methods and materials provided to allow replication by others?

Yes

Is the rationale for creating the dataset(s) clearly described?

Yes

Are the datasets clearly presented in a useable and accessible format?

Yes

Are the protocols appropriate and is the work technically sound?

Yes

Reviewer Expertise:

Bivalve taxonomy, Bivalve transmissible neoplasias, Genome assembly, transcriptomics, molecular cytogenetics and Karyotyping.

I confirm that I have read this submission and believe that I have an appropriate level of expertise to confirm that it is of an acceptable scientific standard.
